# The Role of Protein SUMOylation in the Pathogenesis of Atherosclerosis

**DOI:** 10.3390/jcm8111856

**Published:** 2019-11-02

**Authors:** Sajad Dehnavi, Mahvash Sadeghi, Peter E. Penson, Maciej Banach, Tannaz Jamialahmadi, Amirhossein Sahebkar

**Affiliations:** 1 Department of Immunology, Faculty of Medicine, Ahvaz Jundishapur University of Medical Sciences, Ahvaz 6135715794, Iran; dehnavis1@mailfa.com; 2 Department of Immunology, Faculty of Medicine, Mashhad University of Medical Sciences, Mashhad 9177948564, Iran; sadeghim1@mailfa.com; 3 School of Pharmacy and Biomolecular Sciences, Liverpool John Moores University, Liverpool L3 3AF, UK; p.penson@ljmu.ac.uk; 4 Department of Hypertension, WAM University Hospital in Lodz, Medical University of Lodz, Zeromskiego 113, 93-338 Lodz, Poland; maciej.banach@icloud.com; 5 Polish Mother’s Memorial Hospital Research Institute (PMMHRI), 93-338 Lodz, Poland; 6 Halal Research Center of IRI, FDA, Tehran, Iran; jamialahmadit@mailfa.com; 7 Department of Nutrition, Faculty of Medicine, Mashhad University of Medical Sciences, Mashhad 9177948564, Iran; 8 Biotechnology Research Center, Pharmaceutical Technology Institute, Mashhad University of Medical Sciences, Mashhad, Iran; 9 Neurogenic Inflammation Research Center, Mashhad University of Medical Sciences, Mashhad, Iran; 10 School of Pharmacy, Mashhad University of Medical Sciences, Mashhad 9188617871, Iran

**Keywords:** small ubiquitin-like modifier (SUMO), SUMOylation, atherosclerosis

## Abstract

Atherosclerosis is a progressive, inflammatory cardiovascular disorder characterized by the development of lipid-filled plaques within arteries. Endothelial cell dysfunction in the walls of blood vessels results in an increase in vascular permeability, alteration of the components of the extracellular matrix, and retention of LDL in the sub-endothelial space, thereby accelerating plaque formation. Epigenetic modification by SUMOylation can influence the surface interactions of target proteins and affect cellular functionality, thereby regulating multiple cellular processes. Small ubiquitin-like modifier (SUMO) can modulate NFκB and other proteins such as p53, KLF, and ERK5, which have critical roles in atherogenesis. Furthermore, SUMO regulates leukocyte recruitment and cytokine release and the expression of adherence molecules. In this review, we discuss the regulation by SUMO and SUMOylation modifications of proteins and pathways involved in atherosclerosis.

## 1. Introduction

The development and rupture of atherosclerotic plaques is the most important cause of major clinical cardiovascular events including myocardial infarction and stroke, which were responsible for one-quarter of the deaths worldwide in 2010 [1,2,3]. Atherosclerosis is a chronic, inflammatory, slowly progressing disorder of the arterial wall, characterized by the accumulation of lipids in the walls of large and medium-sized arteries and infiltration of immune cells, leading to the development of atherosclerotic plaques [2,4]. Atherosclerosis can develop asymptomatically for decades until rupture or erosion of the plaque occurs, resulting in intravascular thrombosis, ischemia, and necrosis [5].

Numerous studies have demonstrated that high circulating concentrations of low-density lipoproteins (LDL) and the cholesterol carried within this lipoprotein accelerate the formation of atherosclerotic plaques [6]. Dyslipidemia and an imbalance in the influx and efflux of cellular cholesterol leads to accumulation, oxidation, and finally deposition of LDL and LDL-like particles in the vascular wall and is associated with endothelial cell activation in sites prone to atherosclerotic lesions [7,8,9,10]. Endothelial cell dysfunction occurs commonly in regions of the vessel that experience turbulent blood-flow. Plaque formation is associated with increased vascular permeability, altered composition of the extracellular matrix, and retention of LDL in the sub-endothelial space [6]. Activated endothelial cells up-regulate the expression of cell adherence molecules and release chemokines, leading to the recruitment and infiltration of immune cells, especially circular monocytes and T-lymphocytes [6,7,11,12,13]. LDL particles that become trapped in the intima of the vessel are modified by lipase and protease enzymes and oxidized by myeloperoxidase, lipoxygenase, and reactive oxygen species. They are then taken up by monocytes via scavenger receptors and by resident macrophages in the arterial lumen. As cholesterol esters accumulate as droplets in macrophages, these cells become “foam cells” that are a hallmark of the early stages of plaques [4,6,11,14,15,16]. Oxidized LDL (oxLDL) is a potential damage-associated molecular pattern (DAMP) and induces an inflammatory response.

Foam cells secrete inflammatory cytokines (interleukins and TNF-α) and chemokines (for example, monocyte chemoattractant protein-1 or MCP-1), and these events lead to exacerbation of the local inflammatory response. As the disease progresses, smooth muscle cells migrate to the intima of the affected vessels and initiate the secretion of extracellular matrix components that result in the formation of a fibrotic cap. Symptomatic manifestations of atherosclerosis begin when the fibrotic cap impedes blood flow, leading to angina, myocardial infarction, and stroke [4,17].

Multiple risk factors and pathological stimulants are involved in the pathogenesis of atherosclerosis, including hemodynamic forces, classical cardiovascular risk factors (hyperlipidemia, hyperglycemia and psychologic stress), smoking, diet, environmental factors, and genetics [18,19]. Epigenetics is the study of heritable chromatin modifications that control gene expression by changing the accessibility of DNA or chromatin structure [3,20]. Epigenetic changes, including post-translational modification of nuclear proteins, are involved in the initiation and progression of atherosclerosis. These modifications are dynamic and reversible and are potential targets for anti-atherosclerotic drugs.

## 2. Small Ubiquitin-Like Modifier (SUMO) Proteins and the Process of SUMOylation

Post-translational modifications (PTMs) are covalent, enzymatically catalyzed, modifications of proteins that result in functional changes. To date, more than 200 types of PTMs have been described in eukaryotes and prokaryotes, including ubiquitination, SUMOylation, phosphorylation, glycosylation, acetylation, and methylation [21,22,23,24,25].

PTM by SUMOylation is a critical regulator of the molecular, cellular and systemic functions of proteins. Small ubiquitin-like modifier (SUMO) protein is a member of the ubiquitin-like (Ubl) family with a molecular weight of 10KDa and four isoforms (1, 2, 3, and 4) in humans [26,27,28,29]. SUMO1 and SUMO2 have 50% sequence identity, whereas SUMO2 and SUMO3 have 97% similarity. Types 1, 2, and 3 are expressed ubiquitously, but SUMO4 is only found in the kidney, lymph nodes, and spleen [29,30,31,32,33].

**Figure 1:**
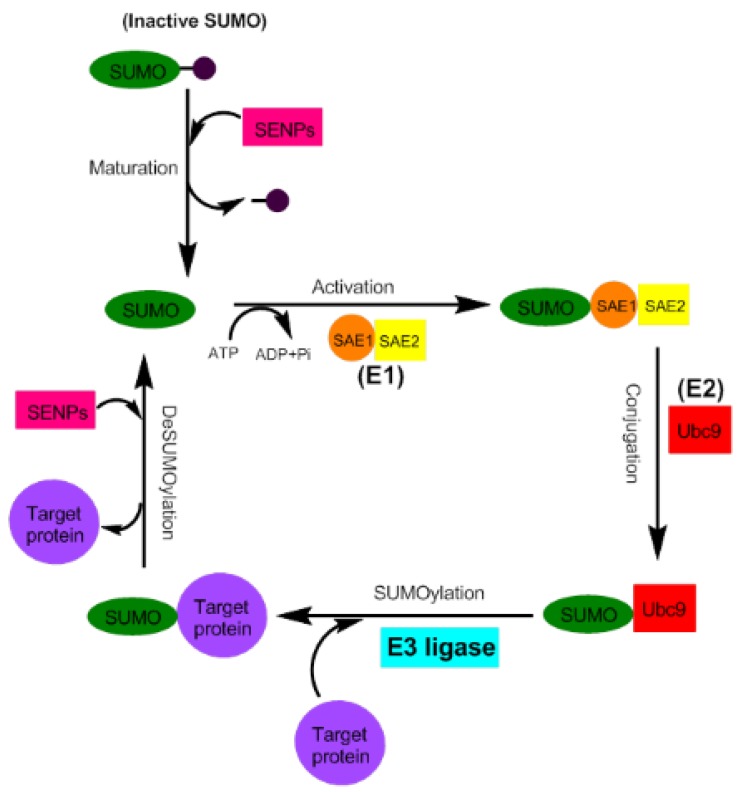
Schematic summary of the SUMOylation pathway. The precursor of small ubiquitin-like modifier (SUMO) is matured by a member of the sentrin-specific protease (SENP) family. This form has a C-terminal di-glycine motif. SUMO is then activated by E1 enzyme, a heterodimer of Aos1 and Uba2. Activated SUMO is passed to the active site of E2 conjugation enzyme, Ubc9. Finally, SUMO is transferred with E3 ligase to the target protein. DeSUMOylation of the substrate is caused by SENP family proteases.

The binding of SUMO to the target substrate is similar to the ubiquitination process and occurs via three distinct steps. First, attachment of SUMO to the E1 activating enzyme (SAE1/SAE2) in an ATP-dependent reaction leads to its activation. Then, activated SUMO is transferred to Ubc9, which is a member of the family of conjugating enzymes known as E2. Finally, Ubc9 targets the SUMO resulting in binding to the specific substrate that is covalently attached to an E3 ligase (PIAS (protein inhibitor of activated STAT), RanBP2 (Ran-binding protein 2), Pc2 (polycomb protein 2)). SUMOylation is a dynamic and reversible process, and SUMO-specific peptidase enzymes (SENP 1, 2, 3, 5, 6, 7) catalyze the deSUMOylation reaction that detaches the SUMO that bond to the lysine residue of target proteins [28,30,34,35,36,37,38] (Figure 1).

SUMOylation can alter protein conformation and influence the surface interactions of target proteins. SUMOylation affects cellular functionality and regulates various cellular processes including intracellular trafficking, subcellular localization, protein–protein interactions, cell cycle progression, DNA repair and replication, RNA metabolism, apoptosis, protein stability, and the activity of kinases [26,28,32,39,40,41,42].

## 3. Blood Flows and Atherosclerosis

The inner surface of blood vessels is lined by a monolayer of endothelial cells that are constantly exposed to flow-derived forces including frictional force (hemodynamic shear stress), stretch, and hydrostatic pressure [43,44,45,46,47]. In large arteries, atherosclerotic plaques predominantly occur in regions in which blood flow is slow or disturbed. This localization suggests that local blood flow patterns have a crucial role in the formation and progression of atherosclerotic plaques. It has been demonstrated that different flow-patterns can modulate the structure and function of endothelial cells (ECs) and regulate the activation of pro-inflammatory and pro-apoptotic genes in ECs [17,47,48,49].

Blood-flow patterns include steady laminar flow (s-flow) and disturbed flow (d-flow). S-flow stimulates ECs to secrete factors that are inhibitors of coagulation, leukocyte inflammation, and smooth muscle cell proliferation as well as promotion of ECs survival [43,50]. Based on the observation that atherosclerotic plaques are rare in regions exposed to s-flow, it seems that this pattern of flow is anti-atherogenic. On the other hand, d-flow induces inflammation, apoptosis, proliferation of ECs and limited vascular reactivity. Most atherosclerotic plaques are located in regions of the arteries exposed to d-flow, such as curved regions. Because d-flow is pro-atherogenic and s-flow is anti-atherogenic, an understanding of molecular signalling pathways which induces by different types of flow and influences on ECs are critical. For example, long-term stable flow up-regulates athero-protective and antioxidant genes such as KLF2, eNOS. Conversely, d-flow induces apoptosis, pro-inflammatory genes, and results in hotspots of ECs with enhanced permeability and inflammation [51,52,53,54].

## 4. NFκB Is a Regulator of Inflammation in ECs and a SUMOylation Target

EC dysfunction leads to pathological inflammation, apoptosis and enhanced cell permeability. Atherogenesis occurs as a result of leukocyte recruitment. The release of cytokines and the expression of adherence molecules are regulated by NFκB [28]. The NFκB family is a group of five transcription factors: p65 (relA), relB, c-Rel, p100/p52, and p100/p52. In its inactive state, NFκB is found in the cytoplasm bound to an inhibitor of κB (IκB). Activation of NFκB occurs when IκB kinase (IκK) is stimulated by an inflammatory factor (such as cytokines or d-flow) IκK phosphorylates IκB and leads to its inactivation via ubiquitination tumorigenesis and inflammatory and cardiovascular disorders [55]. The resultant free NFκB translocates to the nucleus and trans-activates pro-inflammatory genes including VCAM-1, E-selectin, IL-8, IL-1, TNF, and ICAM-1 [56].

SUMOylation has been shown to regulate NFκB through a variety of mechanisms. Modification of the NFκB precursor (p100) activates non-canonical signalling pathways [57]. IκK is a complex of two kinases (IκK1 or IκKα and IκK2 or IκKβ) with a subunit called “NFκB essential modulator” (NEMO) or IκKγ. NEMO has no catalytic activity but is essential for IκK activation and IκB phosphorylation. Deletion of NEMO prevents IκK from efficiently phosphorylating IB and activating NFκB signalling [30,58]. Gareus and colleagues have demonstrated that endothelium-specific depletion of NEMO or dominant-negative IκBα inhibits the formation of atherosclerotic plaque. This observation demonstrates the crucial role of the NFκB signalling pathway in the pathogenesis of atherosclerosis [59]. It has recently been reported that modification of IκBα by SUMO1 inhibits its ubiquitination and destruction and down-regulates NFκB activation. In contrast, Modification of IκBα by SUMO2/3 has the opposite effect and leads to the detachment of IκBα from NFκB and activation of this pathway [28,33,60]. Furthermore, investigations by Miyamoto et al. have demonstrated that genotoxic stress-induced NEMO SUMOylation by SUMO1 results in up-regulation of IκK-NFκB. Interestingly, SENP2 deSUMOylating enzyme, which is a transcriptional target downstream of NFκB, provides a negative feedback mechanism for NFκB activation via NEMO deSUMOylation. Additionally, deSUMOylation of NEMO in K277 via SENP6 inhibits TLR-induced inflammation [27,61,62,63,64].

## 5. Other Targets of SUMOylation Involved in the Pathogenesis of Atherosclerosis

### 5.1. MAPK-Activated Protein Kinase-2 (MK2)

MK2 is a pro-inflammatory kinase that increases NFκB activity by blocking the nuclear retention of p38 and inhibiting phosphorylation of mitogen- and stress-activated protein kinase 1 (MSK1). It has been reported that MK2 depletion significantly inhibits TNFα-induced inflammatory responses in ECs and plaque formation in LDLR-/- mice with decreased expression of VCAM-1 and MCP-1. This observation demonstrates that activation of MK2 in ECs is a pro-atherogenic event. SUMOylation of MK2 K339 inhibits its kinase activity, prevents actin filament remodelling and TNFα-mediated ECs migration. While the exact role of SUMO modification of MK2 is unclear, it may be an important regulator of EC function [33,65,66].

### 5.2. ERK5

Mitogen-activated protein kinases (MAPKs) are a family of serine/threonine kinases that regulate various cellular functions such as proliferation, differentiation, survival, and apoptosis. The MAPK family includes four major subfamilies, of which ERK5 is one of the most important [43]. ERK5 (also called MAPK7) has pivotal roles in endothelial hemostasis and has a unique role in protection against atherosclerosis because it is both a kinase enzyme and a transcription co-activator. As with all MAPKs, ERK5 requires phosphorylation on Thr-X-Thr motif for activation and has a trans-activation motif at its C-terminus that regulates anti-inflammatory genes [51,67,68]. EC-specific ERK5 knockout mice show accelerated apoptosis, inflammation, and atherosclerosis plaque formation [69].

Activation of ERK5 occurs in response to s-flow and induction of upstream MEK5α. Once activated, the arginine-rich middle region binds to the PPARγ1hinge-helix and upregulates the transcription activity of peroxisome proliferator-activated receptor γ (PPARγ). Direct association of ERK5 and PPARγ leads to the release of SMRT1, a PPARγ-corepressor that down-regulates PPARγ transcriptional activity. ERK5-PPARγ association mediated-SMRT1 release leads to inhibition of TNF-mediated NFκB activation and consequently to inflammatory responses [70,71,72]. It has also been demonstrated that ERK5 activation increases the transcriptional activity of PPARδ and leads to inhibition of heme oxygenase1-induced inflammatory response. Moreover, MEK5α/ERK5 activation increases the transcriptional activity of myocyte enhancer factor-2 (MEF-2) which is a critical component of the transcriptional system that is required for the regulation of KLF expression and leads to reduced inflammation. The KLF family, especially KLF2 and KLF4, are targets of ERK5 and play an important role in vascular biology including the regulation of EC inflammation, vascular tone, and permeability [30,73,74].

Hyperglycemia, oxidative stress, and d-flow are inducers of ERK5 SUMOylation and lead to dysfunction and inflammation of ECs. Under normal conditions, ERK5 is not SUMOylated, and SENP2 protects ECs by continuously removing this pathologic modification (ERK5 SUMOylation). SENP2 knockdown leads to increased ERK5 SUMOylation which induces apoptosis and inflammation. It is noteworthy that d-flow affects ERK5 SUMOylation without altering the expression of SENP2 [66]. It has been shown that d-flow induces apoptosis and inflammation via upregulation of ROS production. ROS induces endogenous ERK5 SUMOylation at lysine 6 and 22, leading to inhibition of the transcriptional activity of ERK5 in ECs. This SUMOylation-mediated inhibition of ERK5 reduces the s-flow-mediated KLF2 promoter activity and consequently reduces the expression of KLF2 and eNOS in ECs.

Interestingly, SUMOylation-mediated negative regulation of ERK5 transcriptional activity is independent of the phosphorylation and kinase activity of ERK5. Similarly, the kinase activity of ERK5 is not influenced by ERK5 SUMOylation. It has been suggested that SUMOylation of ERK5 and kinase activity are regulated independently of its transcriptional activity [30,33,43].

### 5.3. P90 Ribosomal s6 Kinase (p90RSK)

P90RSK is a well-characterized serine/threonine kinase that is unique in having two different kinase functional domains, one at each end. This enzyme plays an important role in EC dysfunction in cardiovascular disorders induced by diabetes mellitus [69].

**Figure 2:**
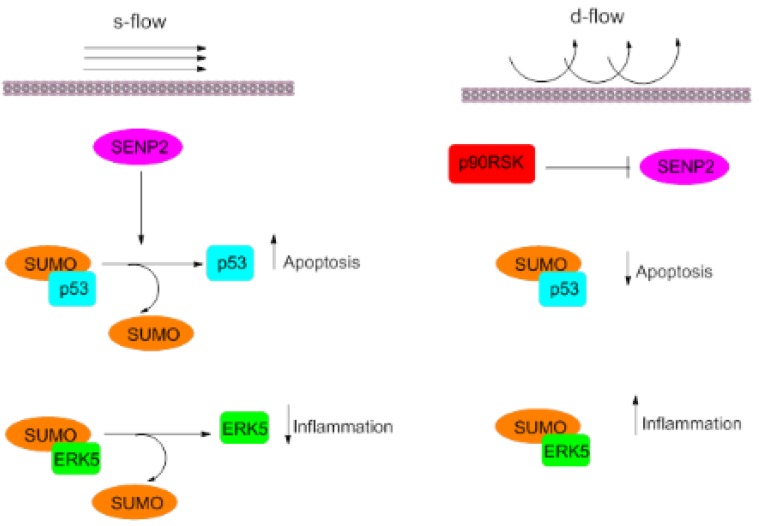
Pro-atherogenic effects of d-flow on inflammation and apoptosis in the p90RSK-dependent pathway. In the steady-state (s-flow), SENP2 enzyme deSUMOylate p53, and ERK5, which are key regulators of inflammation and apoptosis in ECs. Activation of p90RSK, as a result of disturbed flow (d-flow) leads to SENP2 enzyme inhibition. p53 and ERK5 remain in the SUMOylated form, which exacerbates inflammatory conditions and promotes cell proliferation following a decrease in the rate of apoptosis.

P90RSK specifically binds to the C-terminus of ERK5 and phosphorylates the S496 residue, leading to activation of ECs and vascular reactivity and consequently increased expression of VCAM-1 and decreased eNOS. Additionally, p90RSK can modulate ERK5 SUMOylation in a SENP2-dependent pathway. Activation of p90RSK via d-flow leads to SENP2-T368 phosphorylation and nuclear export, which causes a reduction of the deSUMOylating activity of SENP2 and increases SUMOylation of p53 and ERK5 in the nucleus. Consequently, up-regulation of apoptosis and inflammation occur [45,47,52] (Figure 2). Inhibition of the transactivation activity of ERK5 leads to reduced expression of anti-inflammatory genes and causes ECs dysfunction. ECs-specific p90RSK overexpression has been shown to initiate ECs dysfunction and plaque formation in mouse aorta, but this does not occur in dominant-negative (DN)-p90RSK mice. Furthermore, SENP2 depletion in DN-p90RSK mice changes the athero-protective phenotype [47].

## 6. KLF

The proliferation of vascular smooth muscle cell (VSMC) is promoted by cyclin-mediated cell cycle progression and is inhibited by CDK inhibitors. This process plays a pivotal role in the pathogenesis of a variety of disorders including atherosclerosis, hypertension, and vascular aneurysms [75]. KLF is a family of transcription factors all contain zinc fingers. One member of the family, KLF4 has a crucial role in promoting the formation of pluripotent stem cells and is emerging as an important player in tumorigenesis and inflammatory and cardiovascular disorders. It has recently been demonstrated that KLF regulates VSMC proliferation and differentiation by multiple mechanisms [76,77,78].

SUMOylation enables KLF4 to have a dual role in transcription regulation in response to environmental and cellular stimulants. Non-SUMOylated KLF4 is associated with p300 and acts as an activator of p21 transcription and a negative regulator of cell proliferation. In response to PDGF-BB challenge, Ubc9 promotes KLF4 SUMOylation. Consequently, co-repressors, including NCoR, HDAC2, and LSD2, are recruited to the p21 promoter. Inhibition of p21 expression leads to the proliferation of VSMCs [79,80].

Conversely, KLF family members, especially KLF2 and KLF4, are targets of ERK5 that play a key role in the regulation of inflammation in ECs and vascular tone and permeability [67,74]. Both KLF2 and KLF4 are highly expressed by ECs and induced by s-flow, but inhibited by d-flow and diabetic mediators such as AGEs and H_2_O_2_. These factors inhibit inflammation by competing with CBP/p300 cofactor for binding sites on NFκB. Down-regulation of NFκB transcription activity by KLF2/4 leads to attenuation of the expression of pro-inflammatory mediators such as cytokines, chemokines and adherence molecules. Moreover, induction of KLF2/4 expression leads to up-regulation of eNOS expression which has vasodilation, anti-inflammatory and anticoagulant effects. ERk5 activation leads to MEF2 transactivation and consequently down-regulates KLF2/4 [73,81,82,83].

## 7. Membrane-Associated Guanylate Kinase with Inverted Domain Structure-1 (MAGI-1)

Membrane-associated guanylate kinase with inverted domain structure-1 (MAGI-1) is a scaffold protein with six PSD95/DiscLarge/ZO-1 domains, one guanylate kinase domain, and two WW (rsp5) domains that is widely expressed in a variety of tissues, although not in skeletal muscle. MAGI-1 is associated with adherence junctions and is required for cell–cell adhesion and maturation of VE cadherin–mediated adherence junctions. Recently, a genome wide-associated study (GWAS) showed an association between the MAGI-1 locus and the severity of a variety of chronic inflammatory disorders [52,84,85,86,87,88].

It has been demonstrated that MAGI-1 can simultaneously modulate EC activation and apoptosis induced by endoplasmic reticulum stress. These are crucial molecular events in atherogenesis and are regulated by MAGI-1-K931 SUMOylation. DeSUMOylation of MAGI-1-K931 is required for nuclear translocation of p90RSK. Consequently, nuclear p90RSK phosphorylates ERK5-S496 and SENP2-T368 and exacerbates cellular inflammation. Depletion of MAGI-1 significantly inhibits d-flow-induced atherogenesis [45,46,47,69].

## 8. p53

p53 tumor suppressor plays an important role in determining whether cells undergo apoptosis or cell cycle arrest in response to a variety of stresses. Elevated nuclear p53 and up-regulation of pro-apoptotic genes and cell-cycle regulators occurs as a cellular response to DNA damage or stress [89,90]. The role of p53 in atherosclerosis and the biology of flow-sensitive ECs is complex. In human atherosclerotic plaques, elevated p53 has been observed in ECs, suggesting that p53 promotes atherosclerosis. Studies on transgenic mice have shown that p53 overexpression leads to inflammation and dysfunction in ECs via down-regulation of the anti-atherogenic factor KLF2.

Interestingly, p53 overexpression does not exacerbate atherosclerosis in transgenic mice [91,92,93]. Another study demonstrated that p53 knockout increases susceptibility to atherosclerosis and decreases vascular cell turnover. These controversial results may reflect different roles of p53 in different cell-types including ECs, smooth muscle cells, and macrophages [94,95,96,97].

In cultured ECs, exposure to d-flow results in the export p53 from the nucleus to the cytoplasm. This process is regulated by PKCζ-mediated p53 SUMOylation. This occurs because d-flow leads to PKCζ activation via induction of reactive oxygen species (ROS) production; consequently, activation of the PKCζ occurs by interaction of its C-terminal kinase domain with RING domain of PIASγ. The RING domain includes the SUMO ligase catalytic site. PIASγ-PKCζ association may increase the enzymatic activity of PIASγ by altering its tertiary structure. PIASγ–PKCζ association up-regulates p53 SUMOylation. SUMOylated p53 is then transported from the nucleus to the cytoplasm [98]. In the cytoplasm, p53 induces apoptosis via direct interaction with Bax and Bcl-2, preventing the anti-apoptotic function of Bax/Bcl-2 and leading to apoptosis of ECs [53,89,99].

It has also been demonstrated that SENPs have a role in the control of d-flow-induced SUMOylation of p53 and ERK5. Six isoforms of SENPs are found in humans, including SENP2. Knockout SENP2 mice experienced accelerated plaque formation and up-regulation of inflammation and apoptosis in ECs. It appears that this deletion leads to elevated p53 and ERK5 SUMOylation [45,100,101].

## 9. Adenosine Monophosphate-Activated Protein Kinase (AMPK)

AMPK is a stress-activated kinase that controls cellular responses to a variety of stresses through regulation of metabolism, protein synthesis, autophagy, and apoptosis. The enzyme is composed of three subunits, including a catalytic (α) and two regulatory (β, γ) subunits. In unstressed cells, AMPK is localized in the cytoplasm, and it translocates to the nucleus after activation. AMPK can inhibit endoplasmic reticulum stress by reducing ROS production and eNOS activation [102,103,104].

The role of AMPK in the regulation of inflammation in ECs and atherogenesis is isoform-dependent. The α1 isoform is pro-inflammatory, and the α2 is anti-inflammatory. Decreased atherosclerotic plaques formation has been reported in AMPKα1^-/-^/ApoE^-/-^ knockouts, whereas it is increased in AMPKα2^-/-^/LDLR^-/-^ [105]. Rubio et al. demonstrated that SUMO2 modification of AMPKα2 activates this enzyme and protects it from being inactivated via ubiquitination. However, SUMOylation inhibits the activity of AMPKα1 [106].

## 10. Liver Receptor Homolog-1 (LRH-1)

LRH-1 or NR5A2, also called fetoprotein transcription factor, is a member of the NR5A subfamily of nuclear receptors (NRs) that are highly expressed in the intestine and liver and that regulate diverse functions from the development of cholesterol to bile acid hemostasis [107]. The transcriptional activity of LRH-1 is governed by multiple factors, including ligand binding and PTMs, which together dictate the interaction of the receptor and transcriptional co-regulators [108,109,110]. These receptors are targeted by E3-SUMO ligase for SUMOylation at various lysine residues that affect their transcriptional activity. Modification of this receptor by SUMO1 has been demonstrated at lysine 224 located in hinge region of receptor. SUMOylation of human LRH-1 decreases its transcriptional activity through a mechanism that is not yet fully understood. It has been shown that SUMOylation resulted in sequestration the receptor with promyelocytic leukemia (PML) protein bodies. It has been suggested that that SUMO modification of LRH-1 leads to stable recruitment of transcriptional nuclear receptor corepressor-1 and histone deacetylase 3 (NCoR1/HDAC3) corepressor complex and association to G protein pathway suppressor 2 (GPS2) [111,112,113,114].

Recently, Stein et al. demonstrated that modification of lysine 289 of LRH-1 by SUMOylation plays a key role in the formation of atherosclerotic plaques. LRH-1 K289R mice (mutant lysine 289) experience reduced SUMOylation of LRH-1. Loss of SUMOylation in K289 is sufficient for cholesterol transport and protection of mice against atherosclerosis [108].

## 11. Conclusions

Several studies have shown that epigenetic modifications contribute to the initiation and progression of atherosclerosis. Among these modifications, SUMOylation and SUMO protein modifications play important roles in the pathogenesis of atherosclerosis. SUMOylation affects various proteins and pathways including NFκB, ERK, p53, p90RSK, ERK, KLF, MK2, MAGI-1, AMPK, and LRH-1 which are pivotal regulators of the function of ECs (Table 1). Thus, dysregulated SUMO contributes substantially to the development of atherosclerosis. The development of research tools to study SUMOylation and the increasing number of in vivo and in vitro studies in this area have expanded our understanding of the role of the SUMOylation in these pathways. The crucial role of SUMO and SUMOylation in the formation of atherosclerotic plaques presents a novel therapeutic target in the management of atherosclerosis. Further work is required to develop and evaluate therapeutic agents that modulate SUMOylation and that can be used in the prevention and management of atherosclerosis.

## Figures and Tables

**Table 1:** Summary of factors that affected by SUMO modifications and are involved in atherosclerosis pathogenesis.

Protein	General Characteristics and Association with Pathogenesis of Atherosclerosis	Consequences of SUMO Modifications	Ref
NFκB	A family of transcriptional factors localized in the cytoplasm in the inactive form that require stimulation by an inflammatory factor to enable nuclear localization and subsequent activation of pro-inflammatory genes involved in atherogenesis	Down-regulation of NFκB pathway by SUMO1 modification of IκBα Up-regulation of NFκB pathway by SUMO2/3 modification of IκBα	[28,56,57]
MK2	A pro-inflammatory kinase that increases NFκB activity	Inhibition of its kinase activity by K339 SUMOylation	[33,66,67]
ERK5	A family of serine/threonine kinases that regulates various cellular functions especially in endothelial hemostasis and is protective against atherosclerosis	D-flow-induced ERK5 SUMOylation and EC dysfunction	[43,51,67,68,69]
p90RSK	A serine/threonine kinase associated with EC dysfunction in diabetes mellitus-induced cardiovascular disorders	Phosphorylation and consequent inhibition of SENP2 deSUMOylating enzymes which blocks activation of ERK5 and p53 genes	[33,70]
KLF	A family of transcription factors that play an important role in inflammatory and cardiovascular disorders KLF4 is a member of this family which acts as a negative regulator of cell proliferation via p21 interaction in non-SUMOylated state	SUMOylation of KLF4 leads to recruitment of co-repressors to the p21 promoter and increases the proliferation of VSMCs	[68,75,80,81]
MAGI-1	A scaffold protein that is associated with adherence junctions and modulates crucial molecular events in atherogenesis including the activation of ECs and endoplasmic reticulum stress-induced apoptosis.	DeSUMOylation of MAGI-1-K931 is required for nuclear translocation of p90RSK	[52,87]
p53	An important tumor suppressor that plays a role in determining the fate of cell for apoptosis or cell cycle arrest in response to various stresses	SUMOylated p53 exports from nucleus to the cytoplasm and induces ECs apoptosis via direct interaction by pro-apoptotic proteins	[33,90,91,102,103]
AMPK	A stress-activated kinase that orchestrates cellular responses to different stresses	AMPKα2 activation and protection from UQ destruction via SUMO2 modification AMPKα1 inhibition via SUMOylation	[106,107,108,110]
LRH-1	A member of NR5A subfamily for nuclear receptors with various functions such as cholesterol and bile acid hemostasis	Association of K289 LRH-1 SUMOylation and atherosclerosis pathogenesis	[111,112]
